# A systematic review of HIV interventions for black men who have sex with men (MSM)

**DOI:** 10.1186/1471-2458-13-625

**Published:** 2013-07-02

**Authors:** Cathy Maulsby, Greg Millett, Kali Lindsey, Robin Kelley, Kim Johnson, Daniel Montoya, David Holtgrave

**Affiliations:** 1 Department of Health, Behavior and Society, Johns Hopkins Bloomberg School of Public Health, 624 N. Broadway, Baltimore, MD 21205, USA; 2 Division of HIV/AIDS Prevention, Centers for Disease Control and Prevention, 1600 Clifton Rd, Atlanta, GA 30333, USA; 3 National Minority AIDS Council, 1931 13th Street Northwest, Washington, DC 20009, USA

**Keywords:** Black men who have sex with men, HIV, Implementation research, Implementation science, Evaluation

## Abstract

**Background:**

Black men who have sex with men (MSM) are disproportionately burdened by HIV/AIDS. Despite this burden there has been a shortage of research on HIV interventions for black MSM. This article provides a comprehensive review of the literature on interventions for black MSM to identify effective HIV prevention intervention strategies for black MSM.

**Methods:**

We searched 3 databases: *Pubmed*, *Scopus*, and *Google Scholar* to identify peer-reviewed articles and used the following search terms: African American or black; MSM or men who have sex with men and women (MSMW); HIV; program or intervention; and evaluation or intervention science or implementation research. We included research articles that assessed interventions for black men who have sex with men. We included studies that used an experimental, quasi-experimental, or pre-post test design as well as formative research studies. We also searched the CDC and NIH websites to identify planned and on-going intervention studies. We identified a total of 23 studies to include in the review.

**Results:**

We identified 12 completed studies of interventions for black MSM. Eight of these 12 interventions aimed to reduce HIV risk behaviors and 5 found a significant reduction in HIV risk behavior over time. We identified 4 health service intervention studies for young black MSM.

**Conclusions:**

Behavior change interventions are effective at reducing HIV risk behaviors among black MSM. However, relying only on behavioral interventions that aim to reduce HIV risk behavior will most likely not have a population-level effect on HIV infection among black MSM. There is a compelling and urgent need to develop and test comprehensive HIV testing, linkage to care, retention in care and adherence interventions for black MSM.

## Background

Men who have sex with men (MSM) are the group most affected by HIV in the United States and, among MSM, black men are disproportionately burdened. In 2010, black men accounted for 36% of new HIV infections among MSM. The number of new infections among young MSM aged 13-24 increased 22% from 2008 to 2010, with young black MSM accounting for more than half (55%) of new HIV infections among young MSM [1]. Disparities in HIV infection by race among MSM can most likely be explained by a range of complex and interconnected factors including: a greater prevalence of sexually transmitted diseases and unrecognized HIV infection among black MSM; disparities in HIV testing, care and treatment access and use; and social/structural barriers such as income, joblessness, incarceration and discrimination [2-4]. Given the large burden of disease among black MSM, historically there has been a dearth of research on HIV interventions for black MSM [5]. In order to highlight gaps in intervention development and identify effective HIV prevention intervention strategies, this article will provide a comprehensive review of the literature of interventions designed for black MSM. By answering the question “What is the status of HIV prevention intervention research for black MSM”, we aim to inform future research and the development of evidence-based programs.

## Methods

We searched 3 databases: *Pubmed*, *Scopus*, and *Google Scholar* to identify peer-reviewed articles. We conducted this electronic database search on May 16, 2012 using the following search terms: African American or black; MSM or men who have sex with men and women (MSMW); HIV; program or intervention; and evaluation or intervention science or implementation research. We included research articles published in the peer review literature and excluded dissertations, editorials, letters and commentaries. We included HIV interventions that were designed for black or African American MSM. Because of the limited research on this topic, we included studies that used an experimental, quasi-experimental, or pre/post test design, as well as formative research that focused on intervention development. We excluded studies that were not conducted in the United States. Data from each study were extracted, organized into summary tables, and discussed by a team of two reviewers. Disagreements were resolved by discussion and consensus. Summary tables highlighted sample population, sample N, primary outcome, study design, key findings and biases. In addition, to give an accurate description of planned and on-going intervention research for black MSM, we searched the National Institute of Health’s (NIH) RePORTER and clinical trials database as well as the Center for Disease Control’s (CDC) website to identify interventions that met our criteria that were being tested at the time this manuscript was written.

## Results

**Figure 1:**
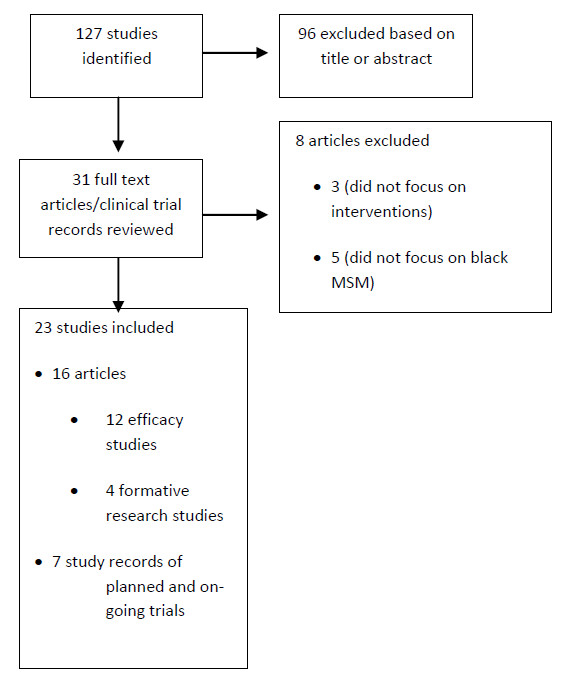
Flow chart: identification and evaluation of studies for systematic review.

Our search yielded 127 records. Of these, 96 were excluded based on their title and abstract because they were clearly not relevant to our review. A total of 31 studies were found to be potentially relevant and the full text of these articles or records was retrieved. Of these, 8 were excluded. Three peer articles were excluded because they did not include an analysis of an intervention and 5 peer articles were excluded because the interventions assessed were for MSM regardless of race. A total of 23 studies were included in this review. Sixteen of these studies had findings published in the peer literature and seven were on-going active studies at the time this review was written (Figure 1).

### Interventions for black MSM

Our literature review identified 12 efficacy studies of interventions for black MSM from the peer-reviewed literature. Eight of these aimed to reduce HIV sexual risk behaviors and 4 focused on use of HIV related medical services. Table 1 provides a brief description of these interventions, including the sample, the location for the study, the intervention name, the theory used to guide the intervention, a description of intervention activities, the study design used, the key outcome measured, key findings, and study limitations.

**Table 1:** HIV Interventions for black MSM

**First author**, **year**	**N total sample**	**Location**	**Intervention name**	**Theory**	**Intervention description**	**Study design**	**Primary outcome**	**Findings**	**Limitations**
Hightow-Weidman (2011)	81 young MSM of color	North Carolina	STYLE (Strength through Youth Livn’ Empowered)	NA	Linkage-to-care program that provided: social marketing campaign, intensive outreach, and a network of medical-social support	Pre- and post- test (with a comparison group)	Viral suppression, mean and median change in CD4, and retention in care (1 appointment over a 4 month period)	1.76% viral suppression at 12 months 2. 101 was the mean change in CD4 over the course of the study 3. OR for clinic visit STLYE cohort vs. pre-STYLE cohort was 2.58 (95% CI 1.34-4.98)	Comparison group not randomized, rather matched on race and age. Comparison group attended clinic prior to STYLE thus temporal effect might be due to differences between groups. Limited data available on comparison group so could not control for some variables that might have been associated with retention. Survey was self-report and administered face-to-face which might have introduced bias. Convenience sample used which may limit generalizability.
Koblin (2011)	283 black MSM	New York City, NY	DiSH	Social Cognitive Theory	Five two-hour group sessions over two weeks. Sexual risk reduction information and exercises integrated into cooking classes	Randomized comparison group	Unprotected anal and receptive intercourse	1. Declines from baseline to 3 months in percentage reporting UIAI, URAI, UIAI with unknown/serodiscordant partners and URAI with unknown/serodiscordant partners for all participants, No significant differences in outcomes by control arm.	Data were self-report and could be subject to social desirability bias and recall bias. Sample size was too small to conduct subgroup analyses. Convenience sample used which may limit generalizability.
Wohl (2011)	61 young MSM of color	Los Angeles	NA	NA	Youth-focused case management with weekly sessions for two months and monthly sessions for the following twenty-two months	Pre- and post- test	Retention in care (2 or more HIV care appointments in the past 6 months)	1. 90% participants retained in care at 3 months and 70% at 6 months 2. Among 33 participants with previous intermittent care, percentage attending clinic visits rose from 7% to 73% over 6 months (p<0.0001). 3. Retention in HIV care at 6 months associated with increased number of intervention visits (UOR 10.5 (95% CI 1.1-96.6, p=0.038).	No control group for this study, rather participants acted as their own controls for pre and post analyses. Pre/post designs suffer from weak internal validity. Small sample size limited ability to calculate OR estimates. Convenience sample used which may limit generalizability.
Wu (2011)	68 black MSM	New York	Connect with Pride	Social Cognitive Theory	Ninety minute intervention sessions delivered to a couple that focused on HIV transmission and risk, social and regulatory skills building, condom use and harm reduction.	Pre- and post-test	Unprotected anal intercourse	1. Baseline to follow-up participants reported significantly fewer sexual partners (mean 4.5 vs 1.6), episodes of UAI (mean 13.3 vs 2.2), and greater condom use with main partners (mean proportion 18% vs 72%)	No control group for this study, rather participants acted as their own controls for pre and post analyses. Pre/post designs suffer from weak internal validity. Small sample size limited ability to calculate OR estimates. Data were self-report and could be subject to social desirability bias and recall bias. However, data were collected using ACASI which should reduce social desirability bias. Convenience sample used which may limit generalizability.
Mangus (2010)	224 young MSM of color	National	Special Projects of National Significance (SPNS)	NA	Enhanced linkage to care projects across eight project sites.	Pre- and post-test	Missed visits	1. Receipt of any program services was negatively associated with missed visits (AOR 0.16 (95% CI 0.03-0.92)	No control group for this study, rather participants acted as their own controls for pre and post analyses. Pre/post designs suffer from weak internal validity. Data were self-report and could be subject to social desirability bias and recall bias. Convenience sample used which may limit generalizability
Operario (2010)	36 young black MSM	Oakland, CA	The Bruthas Project	Information-motivation-behavioral skills model, AIDS Risk Reduction Model	Four one-hour one-on-one HIV counseling sessions. Topics included: general HIV risk reduction counseling, partner dynamics with women, partner dynamics with men, triggers for unsafe sex, and risk reduction goal setting	Pre- and post-test	Unprotected anal intercourse	1. Significant reduction in UIAI (58.3% vs. 33.3%, p=0.02), URAI (44.4% vs. 22.2% (p=0.04), mean number female partners unprotected sex (3.5 vs. 1.7, p=0.01), mean number male partners unprotected sex (1.8 vs. 0.9, p=0.02), sex under influence drugs (86.1% vs. 52.8%, p=0.00) 2. Increase in mean social support (2.9 vs. 3.4, p=0.02), mean self-esteem (3.7 vs. 4.1, p=0.00), loneliness (2.2 vs. 1.0, p=0.02)	No control group for this study, rather participants acted as their own controls for pre and post analyses. Pre/post designs suffer from weak internal validity. Data were self-report and could be subject to social desirability bias and recall bias. However, data were collected using ACASI which should reduce social desirability bias. Convenience sample used which may limit generalizability. High rates of attrition.
Outlaw (2010)	96 black MSM	Detroit, Michigan	NA	Self-Perception Theory, Decision Balance Theory, Trans-theoretical Model	Thirty minute field outreach session with peers using motivational interviewing	Randomized comparison group	HIV counseling and testing & returning for test results	1. Intervention group more likely to receive HIV counseling and testing (49% vs. 20%, p=0.000) 2. Intervention group more likely to return for their test results (98% vs. 72%, p=0.001)	Convenience sample used which may limit generalizability. Peer outreach workers were not blinded to treatment condition which could bias results; however this was minimized by the use of objective outcome measures.
Coleman (2009)	82 African American MSM 50+ HIV+	Pennsylvania	NA	Social Cognitive Theory, Theory of Reasoned Action, and Theory of Planned Behavior	Four two-hour sessions. Topics included: stigma, alienation, co-morbidities associated with aging and HIV, and condom negotiation.	Randomized comparison group	Consistent condom use	1. Risk reduction group OR 2.04 (95% CI: 0.48-8.77, p=0.336) times as likely report consistent condom use in the past 3 months. 2. Among men who did not report consistent condom use at baseline, risk reduction group OR 5.18 (95% CI: .97-27.78) times more likely to report consistent condom use at 3 months.	Convenience sample used which may limit generalizability. Data were self-report and could be subject to social desirability bias and recall bias. Intervention used a female facilitator which might have increase social desirability bias.
Wilton (2009)	338 black MSM	New York City, NY	Many Men, Many Voices (3MV) (DEBI)	Social Cognitive Theory, Behavior Skills Acquisition Model, Trans-theoretical Model , Decision Balance Model	Six two- to three-hour sessions. Topics included: racism, homophobia, sexual relationship dynamics, risk reduction, intentions to change, capacity to change, partnership selection, communication, negotiation, social support, and problem solving.	Randomized comparison group	Unprotected anal intercourse	1. UAI casual partner case vs. control: RR any UAI at 6 months (0.34 (95% CI 0.14-0.83)) and RR IUAI (0.24 (95% CI 0.09-0.65)) 3. GEE model baseline to 6 months case vs. control: RR UIAI with casual partners (0.49 (95% CI 0.28-0.87))	Waitlist comparison condition was used to evaluate the intervention which might bias the study towards finding significant results. Convenience sample used which may limit generalizability. Data were self-report and could be subject to social desirability bias and recall bias. However, data were collected using ACASI which should reduce social desirability bias.
Williams (2008)	137 HIV positive African American and Latino MSM	Los Angeles	Sexual Health Intervention for Men (S-HIM)	Social Cognitive Theory	Six two hour session intervention. Topics included; gender and ethnicity, early socialization around gender and culture, stigma, problem solving, psychological well-being, recognizing triggers	Randomized comparison group	Unprotected anal intercourse	1. Sample as a whole reported reductions in sexual risk behaviors and number of sex partners from baseline to post-test, and from 3 to 6 months. 2. Decrease in sexual risk behaviors from baseline to post-test only significant for S-HIM participants	Convenience sample used which may limit generalizability. Data were self-report and could be subject to social desirability bias and recall bias.
Jones (2008)	Approximately 300 black MSM	Three cities in North Carolina	d-up: defend yourself (DEBI)	Diffusion of Innovations	Popular opinion leader model. Opinion leaders trained during four two-hour sessions. Topics included: racism, homophobia, bisexuality, employment and poverty, religion, condom use demonstration, and skills building in risk reduction communication.	Repeated cross-section surveys at 4 equally spaced time points	Unprotected anal intercourse	1. Found significant linear trends in decreases in UIAI, URAI, and UAI with male partners at 4, 8, and 12 months 2. At 12 months, mean number of URAI partners decreased by 40.5%, mean number of UIAI episodes decreased by 53.0%, mean number of URAI decreased by 56.8%, percentage condom use UIAI increased by 23.0% and percentage condom use URAI increased 30.3%.	No control group for this study which may weaken internal validity. Data were self-report and could be subject to social desirability bias and recall bias. However, data were collected using self-administered surveys on handheld computers. Convenience sample used which may limit generalizability.
Peterson (1996)	318 African American gay and bisexual men	San Francisco Bay Area	Brother to Brother	Social Cognitive Theory and AIDS Risk Reduction Model	Experimental intervention was either one three-hour session or thee three-hour sessions. Topics included: racial and sexual identity, perceptions of HIV risk, HIV risk education, assertiveness training, and behavioral commitment.	Randomized comparison group	Unprotected anal intercourse	1. Participants in the triple session group reduced UAI from 46% to 20% at 12 months and from 45% to 20% at 18 months 2. Participants in the single session intervention reduced UAI from 47% to 38% at 12 months and 50% to 38% at 18 months 3. Participants in the control group had no change in UAI at 12 months (26% to 23%) and 18 months (24% to 18%)	Convenience sample used which may limit generalizability. Data were self-report and could be subject to social desirability bias and recall bias. High rates of attrition limited ability to accurately detect differences between groups however used an intent-to-treat approach. Selection bias might be present as there were differences between exposure and comparison group at baseline.

### Behavioral risk reduction interventions

Of the 8 interventions that aimed to reduce HIV sexual risk, 5 used randomized comparison group design [6-10], 2 used a pre-post test design [11,12] and one used a repeated cross-sectional design [13]. Seven of the 8 behavioral risk reduction interventions identified in our review provided a series of health education and skills-building sessions. Five of these interventions were in a group setting [6-10], while the BRUTHAS project was comprised of a series of one-to-one counseling sessions [11] and Connect with Pride was couples based [12]. These multi-session interventions addressed topic areas such as condom use, relationship dynamics, skills building around communication and risk reduction. The duration of these interventions varied. For example, Brother to Brother was a three hour intervention while both S-HIM and Many Men, Many Voices included six two-hour sessions.

The one intervention that was not session-based was d-up: Defend Yourself! D-up is a DEBI (Diffusion of Effective Behavioral Interventions) that is based on the popular opinion leader model. This model aims to increase safer sex norms by training popular opinion leaders to have risk-reduction conversations with their friends [13].

All of the behavioral risk reduction interventions targeted black MSM, addressed the social context of HIV risk reduction for black MSM by including discussion in areas such as stigma, racism, masculinity and homophobia, and were grounded in theory. Interventions were tailored for young MSM [13,14], MSM of unknown or negative HIV status [10], older HIV-positive MSM [6], MSM with a history of child abuse [9], men who have sex with men and women [11], and MSM that use methamphetamines [12].

Seven of the behavioral interventions assessed unprotected anal intercourse as their primary outcome of interest [7-13] and one assessed consistent condom use [6]. Of the 8 behavioral interventions identified by this review, 5 were found to reduce HIV sexual risk behavior. The evaluation of d-up! measured unprotected receptive and insertive anal intercourse at 4 months, 8 months, and 12 months. The study found reductions at all time points. At 12 months, the study found 31.8% reduction in any unprotected anal intercourse as well as a 40.5% reduction in mean number of unprotected receptive anal sex partners [13]. Peterson found that Brother to Brother participants who engaged in a triple session intervention reduced frequency of unprotected anal intercourse from 45% (95% CI 0.33-0.57) at baseline to 20% (95% CI 0.12-0.31) at 18 months. Participants who engaged in a single-session intervention saw only slight decreases in unprotected sex from baseline to 18 months (50% (95% CI 0.37-0.63) to 38% (95% CI 0.26-0.51). Among the control group, frequency of risky sex remained constant over time, 24% (95% CI 0.14-0.38) at baseline versus 18% (95% CI 0.09-0.32) at 18 months. These results suggest the superiority of the triple session over the single session in reducing risky sexual behavior [8]. Wilton found that *Many Men*, *Many Voices* participants, when compared to controls, had significant reductions in mean number of any unprotected anal intercourse episodes with casual partners (RR=0.34; 95% CI=0.14-0.83) at 6 months and male sex partners (RR=0.75; 95% CI=0.57-0.98) at 3 months [10]. At 3 months, the BRUTHAS project found significant reductions in unprotected receptive (44.4% versus 22.2%, *p*=0.04) and insertive anal intercourse (58.3% versus 33.3%, *p*=0.02), as well as reductions in mean number of female (3.5 versus 1.7, *p*=0.01) and male (1.8 versus 0.9, *p*=0.02) unsafe sex partners and sex under the influence of drugs (86.1% versus 52.8%, *p*=0.00). The BRUTHAS project also found increases in mean social support (2.9 versus 3.4, *p*=0.02) and mean self-esteem (3.7 versus 4.1, *p*=0.00) and decreases in mean loneliness (2.2 versus 1.9, *p*=0.02) [11]. Connect with Pride found that at 2 months follow-up, study participants reported significantly fewer sexual partners (mean 4.5 versus 1.6) and mean number of episodes of UAI with main partners (13.3 versus 2.2) [12].

Of the 8 behavioral risk reduction interventions included in this review, 3 had inconclusive findings. S-HIM found a reduction in sexual risk behavior for the whole sample from baseline to post-test and from 3 to 6 months and no significant main effect of condition on level of sexual behavior. The study also found that there was no main effect of condition on number of sex partners or depression [9].

The evaluation of a HIV risk reduction intervention for older HIV seropositive black men found that MSM who were exposed to the intervention were equally likely to use condoms consistently compared to controls (OR=2.04; 95% CI=0.48-8.77, *p*=0.336). However, among men who did not report consistent condom use at baseline, exposure to the intervention was marginally associated with an increase in consistent condom use at 3 months (OR 5.18; 95% CI=0.97-27.78, *p*=0.054) [6].

The DiSH study found significant decreases among all participants from baseline to 3 months in unprotected insertive anal intercourse (UIAI), unprotected receptive anal intercourse (URAI), UIAI with unknown or serodiscordant partners, and URAI with unknown or serodiscordant partner (p<.0001 for all). However, the study arm did not differ significantly from the control arm for these outcomes. The study also found declines for all participants from baseline to 3 months in number of male partners and mean number of unprotected anal intercourse partners and increases in condom use. Again, these findings did not differ significantly by study arm [7].

### HIV service interventions

We identified 4 peer review articles on evaluated health service interventions. A study by Outlaw aimed to increase HIV testing among young black MSM. The study found that men exposed to the motivational interviewing condition were more likely to receive HIV counseling and testing (49.0% versus 20.0%, p=0.000) and that exposed individuals were more likely to return for their test results than men who received standard care (98.0% versus 72.0%, p=0.001) [15]. Motivational interviewing is a client-centered goal-oriented communication method to increase motivation and confidence for behavior change [16].

The Health Resources Service Administration (HRSA) HIV/AIDS Bureau’s Special Projects of National Significance (SPNS) was a national multi site enhanced linkage-to-care program implemented for young MSM (YMSM) of color. A cross-site evaluation of 224 young MSM of color found that enhanced linkage-to care services reduced the odds of having a missed HIV medical visit (AOR=0.16; 95% CI=0.03-0.92). In addition, the evaluation found that feeling respected at the clinic was associated with retention in care [14]. One of the demonstration sites for SPNS, Los Angeles, conducted an evaluation of their case management intervention. The intervention included weekly case management visits for 2 months and monthly visits for the subsequent 22 months. The evaluation used a pre/post test design without a comparison group. The evaluation found that among intermittent care participants (n=33), the average number of HIV care visits increased from 0.2 to 5.5 from baseline to 6 months (*p*=<0.0001) and that 82% of participants who had been in intermittent care were retained in care at 6 months. (Intermittent care was defined as having less than 2 HIV primary care visits in the past 6 months). The evaluation also found a significant dose–response trend between number of hours in the intervention and retention in HIV care (*p*=0.02) [17].

STYLE (Strength Through Youth Livin’ Empowered) is a SPNS intervention conducted in North Carolina that aimed to diagnose, engage, and retain minority young MSM (YMSM) in HIV primary care services. The intervention included a social marketing campaign, outreach to minority YMSM, and the availability of a medical-social support network that included a peer outreach worker, a physician, a case manager, support groups, research staff and local AIDS social service organizations. The intervention used a pre/post test design with a comparison group comprised of clinic participants prior to the start of the STYLE program. The intervention enrolled 81 participants and found that 83% of participants had at least one medical visit every 4 months (retained in care). The odds ratio for attending a clinic visit was 2.58 for the STYLE cohort compared to the pre-STYLE cohort (95% CI 1.34-4.98) [18].

### Intervention development

We identified four formative research articles in the peer-reviewed literature that focused on intervention development (Table 2). An article by Hightow-Weidman details the formative process used to develop HEALTHEMPOWERMENT.org, a theory-based online HIV/STI prevention intervention for young black MSM [19]. Formative research was conducted in three phases. Focus groups, semi-structured interviews and an expert panel contributed to the first version of the site. Two rounds of usability testing resulted in additional versions that included new features such as added intervention components, topic areas, and a community networking function. The formative research found that the development of a culturally meaningful and developmentally appropriate interactive HIV/STI website was a highly iterative process [19].

**Table 2:** Formative research for HIV interventions for black MSM

**First author,year**	**N total sample**	**Location**	**Intervention Name**	**Theory/Model**	**Description**	**Evaluation design**	**Findings**
**Formative Research and Intervention Development**							
Jagantha (2011)	NA	California	Harnessing Online Peer Education (HOPE)	Community Population Opinion Leader Model	Training of peer leaders in HIV prevention education and behavior change to interact with participants on a Facebook group.	Pre-post questionnaire, activity log, content analysis of Facebook discussion, and interviews	Evaluation results not included in article
Hightow-Weidman (2011)	NA	North Carolina	HEALTHMPOWERMENT.ORG	Institute of Medicine’s Integrated Model of Behavior Theory	Formative research for website development	3 focus groups, semi-structured interviews, and two rounds of usability testing	1. Website development was an iterative process 2. Usability testing suggests that site is high rates of satisfaction, content acceptability, and usability
Wu (2010)	8 couples with at least one African American partner per couple	New York	Pilot version of Connect with Pride	Theory of Behavior Change	Formative research for intervention adaptation	6 focus group discussions	1. Developed a relationship-oriented framework for sexual risk behavior for African American, methamphetamine-involved male couples 2. Adapted Couples an intervention for heterosexual couples for African American couples by adapting and revising activities
Williams (2009)	58 African American MSMW	Los Angeles	Men of African Legacy Empowering Self (MAALES)	Theory of Reasoned Action and Planned Behavior, Empowerment Theory, Critical Thinking and Cultural Affirmation Model	Formative research for intervention adaptation	9 focus group discussions, 20 individual interviews, and a pilot intervention session	1. Developed a culturally congruent intervention for African American MSMW 2. Early evaluation and case studies indicate high level of participant satisfaction and favorable outcomes

A study by Williams described the formative research and development process that contributed to the creation of MAALES, an intervention for black MSMW. The intervention was informed by 9 focus groups and 20 one-to-one interviews and included pilot intervention testing. MAALES intervention involves 6 sessions covering topics such as meaning ascribed to an African American male identity, HIV in the African American community, risk assessment, harm reduction, communication skills building, and goal setting. In addition to reducing HIV risk behavior, the intervention was also designed to increase racial and cultural pride. Initial early evaluation activities and case study data from MAALES suggest a high level of participant satisfaction [20].

A paper by Wu describes the formative research findings that were used to inform the adaptation of an intervention for methamphetamine-involved heterosexual couples, Connect, into an intervention for methamphetamine-involved African American MSM couples. A total of 8 couples participated in 6 focus group discussions. The study developed a relationship-oriented framework for methamphetamine-involved African American male couples. The couples intervention was then divided into component activities; each component activity was revised to adhere to the framework and reconstructed. The intervention team then planned to pilot test the revised intervention with a small sample of its target audience [21]. The resulting pilot intervention, Connect with Pride, was discussed in the previous section [12].

A recent article by Jaganatha described the training of peer leaders to initiate HIV discussions on social networking sites. The study, called HOPE (Harnessing Online Peer Education), adapted the community popular opinion leader method of using influential members of the community to motivate behavior change for social networking sites. The study targeted Latino and African American MSM. The peer training included 5 broad topic areas: recruitment, HIV knowledge, social context of stigma and cultural barriers to HIV prevention, communication and the creation of effective health communication messages, and ethical considerations of using social media as health prevention tool [22].

### Planned and on-going research

When we conducted on-line searches of the CDC (cdc.gov ) and NIH (projectreporter.nih.gov/reporter.cfm and clinicaltrials.gov) we identified seven additional studies that are in various stages of implementation. The Safer Sex Program for Young African-American Men is a randomized control trial that will test the efficacy of an intervention to reduce STD and HIV acquisition among young black MSM. This clinic-based study aims to be completed in 2016. Connect’n Unite: Couples-Based HIV/STI Prevention for Drug-Involved, Black MSM (CNU) is a randomized clinical control trial to test the efficacy of the Connect with Pride intervention. This study aims to be completed by 2015. We identified five studies that were actively recruiting at the time this paper was written, three of which focus on MSMW. Project Power is an internet-based intervention for MSMW that includes three two-hour sessions facilitated by health educator on HIV risk reduction. The intervention condition will be compared to control condition that is a one two-hour internet session that aims to improve general health. A Randomized Control Trail of the Bruthas Project is assessing the effect of an individual-level HIV prevention intervention for MSMW. The intervention is delivered in four sessions by trained counselors and covers topics such as HIV transmission, sexual communication skills, and condom use. RISE is an individual-level intervention for black MSMW that uses a randomized comparison group design. RISE involves 6 two-hour coaching interventions with a trained counselor that covers topics such as coping, stress reduction, and sexual health. In addition, MyLife MyStyle Evaluation Project is comparing the effectiveness of the MyLife MyStyle intervention to decrease the occurrence of unprotected anal sex among participants compared to a wait-list control group. Black Men Evolving Behavioral HIV Prevention Intervention for Black MSM (B-ME) is a two and a half day HIV prevention and risk reduction retreat. The study uses a randomized control group design to compare the effects of the intervention arm to a standard of care control arm.

## Discussion

We identified 12 interventions in the peer review literature for black MSM that had been rigorously tested. Seven of these interventions used a randomized comparison group design, the gold standard in study design. We identified 8 HIV prevention interventions for black MSM that reduced unprotected anal intercourse among study participants and 5 of these reached statistical significance. The success of these interventions suggests that behavior change interventions are effective at reducing HIV risk behaviors among black MSM. We were also pleased to identify completed and on-going interventions that are tailored for sub-groups of MSM with unique needs such as MSMW, drug-users, and youth. Interventions that have been shown to effectively reduce HIV risk behaviors should be used to inform evidence-based programs that are implemented on a wider scale and in a diversity of settings.

We also identified four formative research studies. These studies provide valuable information on the process of intervention development and on feasibility testing which can help to inform researchers and practioners as they develop and adapt interventions.

Behavior-change interventions that address areas such as healthy relationships, relationship dynamics, the social context of HIV risk, and risk reduction are valuable for HIV prevention, especially for HIV- positive black MSM. However, the high rates of HIV among black MSM cannot be explained by differences in HIV risk behavior [2]. Therefore, relying solely on behavioral interventions that have an endpoint of reducing risk behavior will most likely not have a population-level effect on HIV infection among black MSM. Research indicates that elevated HIV rates among black MSM can be explained by high rates of undiagnosed seropositivity, high rates of STIs, low use of ART and low ART adherence [2,23,24]. Despite this, we identified a small number of studies of interventions to increase use of HIV testing or treatment services among black MSM. We identified one intervention aimed to increase HIV testing among black MSM as well as SPNS, a national program that included eight linkage-to-care programs for young HIV-positive MSM of color.

To increase the number of seropositive black MSM who are aware of their HIV status and in HIV care and treatment, there is a great need for comprehensive HIV testing, linkage to care, and retention in care intervention studies for black MSM. Studies are needed that operate across various levels, including the health systems level and the individual level. At the health systems level, intervention studies are needed to test the effectiveness of developing well-defined systems that operate across and within organizations working with People Living with HIV (PLWH) to identify, link, and retain black MSM living with HIV into culturally appropriate HIV care and treatment. The SPNS’s enhanced linkage to care program found that feeling respected at the clinic was important for retention in care [14]. Future intervention studies for black MSM should address the health care environment characteristics that promote linkage and retention in care among black MSM. At the individual level, studies are needed to test the effectiveness of interventions that offer outreach, linkage to care, retention, and ART adherence support services to black MSM. Programs such as ARTAS-I and ARTAS-II have shown that brief case management can successfully increase rates of linkage to care [25,26], HIV systems navigation, an adaptation of patient navigation, has been found to successfully reduce barriers to care and improve health outcomes [27], and several adherence interventions, including social support interventions and case management interventions, have been proven effective [28]. These programs could be used to inform the development of intervention studies designed specifically for black MSM.

This review faces many limitations. First, we focused on studies in the peer-review literature and thus excluded research not accepted for publication as well as research in the gray literature. Thus, by focusing our search on studies with published findings we may have biased our results towards studies with significant findings and may have excluded studies with null findings. Second, this review also excludes interventions that have been developed and implemented but not rigorously studied. Evaluation research places an emphasis on internal and external validity [29] and, as a result, it requires methods and resources beyond the scope of many programs. Third, interventions that were not specifically designed for black MSM but which reach black MSM were also excluded. This includes HIV interventions for MSM regardless of race/ethnicity and HIV interventions for black men regardless of sexual orientation as well as broad initiatives around HIV testing, HIV prevention, HIV treatment, and provision of support services such as housing, substance abuse treatment, and job training. Fifth, we searched three databases using specific search terms to identify articles for potential inclusion in this study as well as NIH and CDC websites. While we feel our review was exhaustive, it is possible that we would identify additional articles if we expanded our list of search terms and the databases searched. Sixth, we included evaluations in our review that had a pre/post design and evaluations without a comparison group. These designs are limited in their ability to attribute findings to the intervention being assessed and have weak internal validity. Therefore these results should be interpreted with caution. Seventh, this review is qualitative and does not include a meta-analysis. Finally, we draw conclusions across studies without accounting for moderating factors.

## Conclusion

This article provided a comprehensive review of implementation science among black MSM. We identified a total of 8 sexual risk reduction intervention studies among black MSM. Of these, 5 were found to significantly reduced HIV risk behaviors. Successful HIV behavior risk reduction interventions for black MSM should be used to inform evidence-based programming implemented on a wider scale. We identified no evaluated HIV identification, linkage to care, or retention in care programs for adult black MSM and a limited number for young black MSM. There is an imperative to develop, test, and scale up culturally appropriate HIV testing, linkage, retention, and adherence initiatives for black MSM. To successfully combat HIV/AIDS among black MSM interventions are needed that integrate behavioral, structural, and biomedical components.

## Competing interests

The authors declare that they have no competing interests.

## Authors’ contributions

CM researched, analyzed, interpreted the data, and wrote the manuscript. DH contributed to the design, research, analysis, and interpretation of the findings. GM, KL, RK, KJ, DM critically revised the manuscript for important intellectual content. All authors read and approved the final manuscript.

## Pre-publication history

The pre-publication history for this paper can be accessed here:

http://www.biomedcentral.com/1471-2458/13/625/prepub
